# Formation of autotriploid *Carassius auratus* and its fertility-related genes analysis

**DOI:** 10.1186/s12864-021-07753-5

**Published:** 2021-06-10

**Authors:** Chongqing Wang, Xiang Luo, Huan Qin, Chun Zhao, Li Yang, Tingting Yu, Yuxin Zhang, Xu Huang, Xidan Xu, Qinbo Qin, Shaojun Liu

**Affiliations:** State Key Laboratory of Developmental Biology of Freshwater Fish, Engineering Research Center of Polyploid Fish Reproduction and Breeding of the State Education Ministry, College of Life Sciences, Hunan Normal University, Hunan 410081 Changsha, People’s Republic of China

**Keywords:** Fertility, Autotriploid fish, Transcriptome, Gonad

## Abstract

**Background:**

Formation of triploid organism is useful in genetics and breeding. In this study, autotriploid *Carassius auratus* (3nRR, 3n = 150) was generated from *Carassius auratus* red var. (RCC, 2n = 100) (♀) and autotetraploid *Carassius auratus* (4nRR, 4n = 200) (♂). The female 3nRR produced haploid, diploid and triploid eggs, whereas the male 3nRR was infertile. The aim of the present study was to explore fertility of potential candidate genes of 3nRR.

**Results:**

Gonadal transcriptome profiling of four groups (3 females RCC (FRCC), 3 males 4nRR (M4nRR), 3 males 3nRR (M3nRR) and 3 females 3nRR (F3nRR)) was performed using RNA-SEq. A total of 78.90 Gb of clean short reads and 24,262 differentially expressed transcripts (DETs), including 20,155 in F3nRR vs. FRCC and 4,107 in M3nRR vs. M4nRR were identified. A total of 106 enriched pathways were identified through KEGG enrichment analysis. Out of the enriched pathways, 44 and 62 signalling pathways were identified in F3nRR vs. FRCC and M3nRR vs. M4nRR, respectively. A total of 80 and 25 potential candidate genes for fertility-related in F3nRR and M3nRR were identified, respectively, through GO, KEGG analyses and the published literature. Moreover, protein-protein interaction (PPI) network construction of these fertility-associated genes were performed. Analysis of the PPI networks showed that 6 hub genes (*MYC*, *SOX2*, *BMP4*, *GATA4*, *PTEN* and *BMP2*) were involved in female fertility of F3nRR, and 2 hub genes (*TP53* and *FGF2*) were involved in male sterility of M3nRR.

**Conclusions:**

Establishment of autotriploid fish offers an ideal model to study reproductive traits of triploid fish. RNA-Seq data revealed 6 genes, namely, *MYC*, *SOX2*, *BMP4*, *GATA4*, *PTEN* and *BMP2*, involved in the female fertility of the F3nRR. Moreover, 2 genes, namely, *TP53* and *FGF2*, were related to the male sterility of the M3nRR. These findings provide information on reproduction and breeding in triploid fish.

**Supplementary Information:**

The online version contains supplementary material available at 10.1186/s12864-021-07753-5.

## Background

Polyploid organisms have three or more chromosome sets. Triploidy, an example of polyploids, plays a vital role in the process of biological evolution and can be divided into autotriploidy and allotriploidy [1]. Allotriploids have three chromosome sets from two or more different species, whereas autotriploids have three chromosome sets derived from a single taxon.

Development of gonads is critical to fertility in sexually reproducing organisms especially in triploids and is tightly regulated by complex processes [2]. Sex determination, sexual differentiation and gametogenesis are important processes during gonadal development. Any abnormality in these events can result in infertility. Several genes implicated in sexual determination and differentiation have been reported [3–5]. Gametogenesis, including oogenesis and spermatogenesis, are also regulated by complex mechanisms and several regulatory genes [6, 7]. A previous study explored regulation of early stages of oogenesis [8]. In addition, studies explored biological mechanisms that occur mid-oogenesis [9], and regulation of late oogenesis [10]. Spermatogenesis is divided into three steps: spermatogonial mitotic proliferation, two times of meiosis, and post-meiotic differentiation [11]. Previous studies explored the functional mechanisms of spermatocytogenesis [12], meiosis during spermatogenesis [13], and spermiogenesis [14].

Fertility of polyploids has important implications in fisheries and sustainable aquaculture. Artificial triploids of species such as *Atlantic salmon*, *Oncorhynchus mykiss*, *Salmo trutta* and *Salvelinus fontinalis* have been widely used in fish farming industry [15]. There has been a believe that triploidization causes infertility in fish. In addition, a previous study reports that the triploid can channel the energy required for gonad maturation to somatic growth, causing rapid growth rates compared with their diploid counterparts [16]. However, a different study reported that triploid fish can produce normal gametes [17]. In our previous study, *Carassius auratus* red var. (RCC) (female) and autotetraploid *Carassius auratus* (4nRR) (male) were artificially hybridized to produce hybrid autotriploid *Carassius auratus* (3nRR). After hybridization, the male 3nRR did not produce normal sperm, whereas the females generated dynamic eggs [18]. Analysis of meiosis-related gene expression showed that *Dmc1* and *Ph1* had higher expression level in female 3nRR compared with levels in the males, indicating that these genes are involved in regulating fertility of 3nRR [19]. Molecular mechanisms involving 3nRR fish in controlling fertility have not been explored fully.

RNA-Seq technology is utilised for analysis of the structure and function of genes at the organismal level, and for exploring a series of biological pathways [20]. RNA-Seq technique has been successfully used in studies various fishes in the past decade. In spotted scat species (*Scatophagus argus*), several candidate genes involved in reproduction and gonadal development were obtained by RNA-Seq [21]. Studies on *Takifugu rubripes* reported that sex-related genes play an important role at early sex differentiation stage [22]. Gonadal transcriptome profiling of triploid hybrid loaches (*Misgurnus anguillicaudatus*) and their diploid and tetraploid parents showed key genes implicated in low hybrid triploid fertility [17]. A study on *Thunnus maccoyii* reported sex and gonad-development-related genes in the gonads of Southern bluefin tuna through RNA-Seq [23]. In addition, RNA-Seq has been successfully used to analyze sex determination and differentiation related genes in tilapia [24]. In the present study, we successfully obtained triploid fish (3nRR) by crossing female RCC and male 4nRR. The diploid (2nF_1_), triploid (3nF_1_) and tetraploid (4nF_1_) hybrids were then generated by hybridization of female 3nRR and male RCC. In this study, we explored important biological traits and systematically compared gonadal transcriptome of the triploid fish (3nRR) with their parents. Further, the molecular mechanism of the low fertility of the autotriploid fish was explored. The findings of this study provide information on the biological characteristics of 3nRR and mechanisms associated with fertility regulation in triploid fish.

## Results

### Fertility of autotriploid *Carassius auratus*

**Fig. 1:**
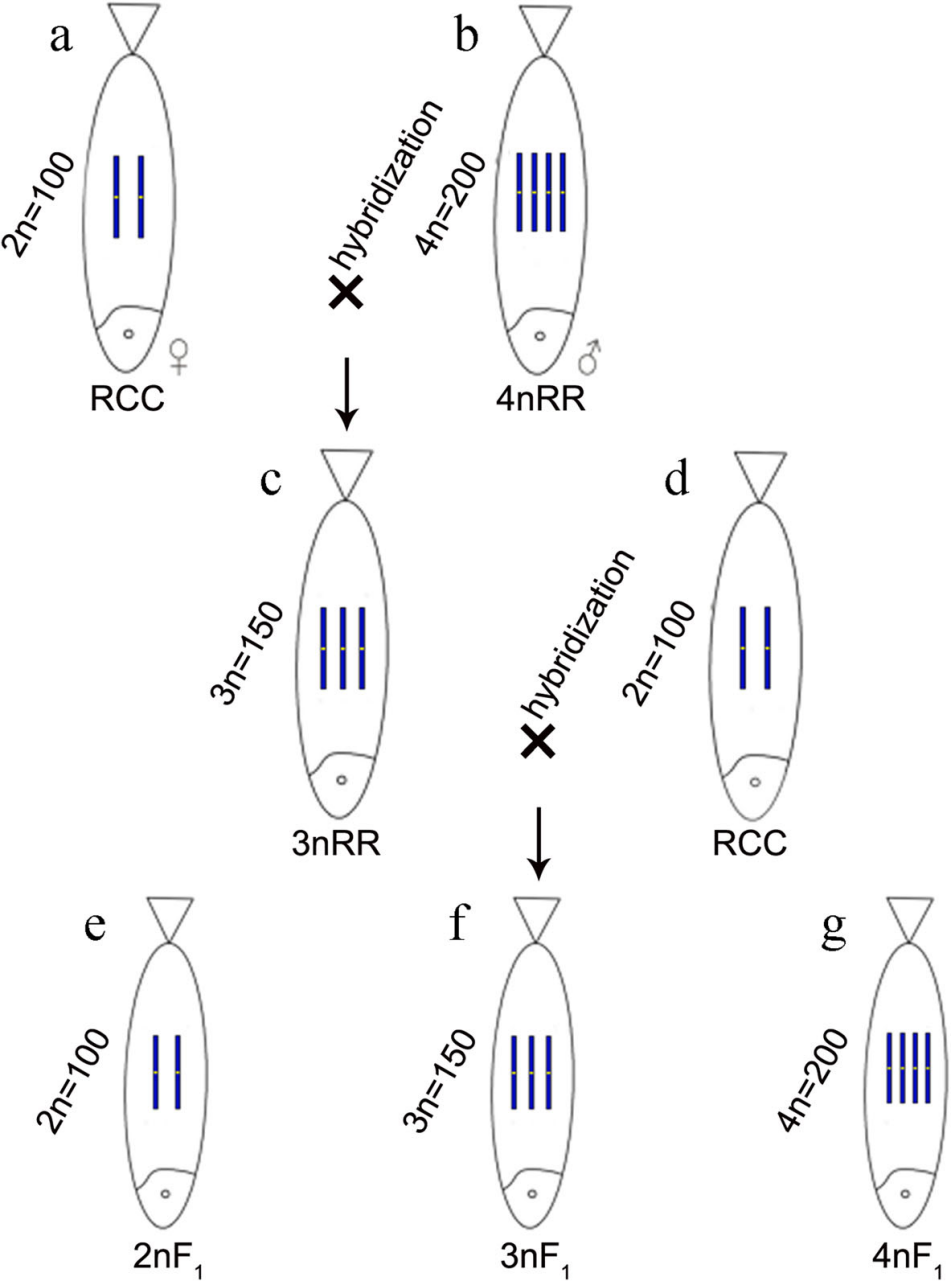
Formation of polyploid fish

**Fig. 2:**
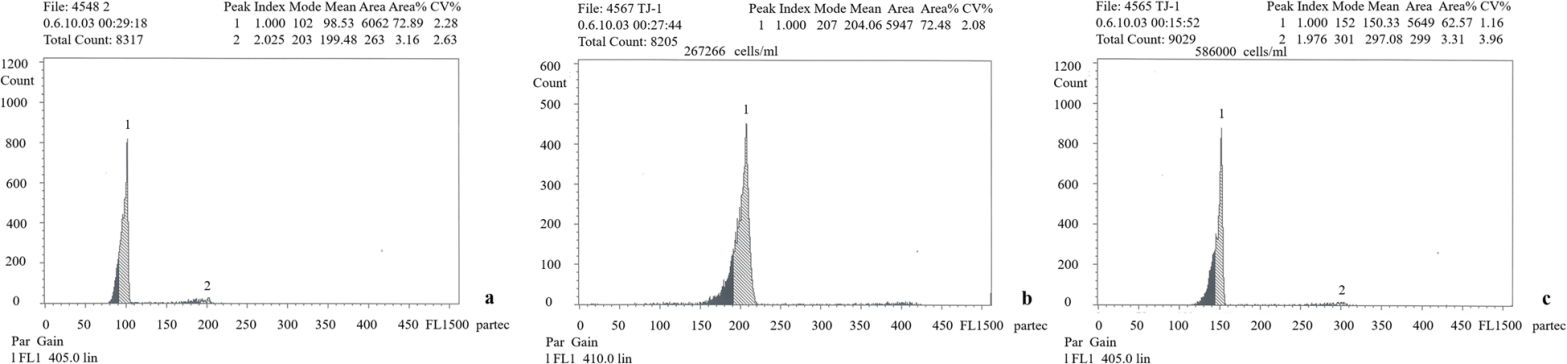
DNA-content flow-cytometry histograms of RCC (**a**), 4nRR (**b**) and 3nRR (**c**)

**Fig. 3:**
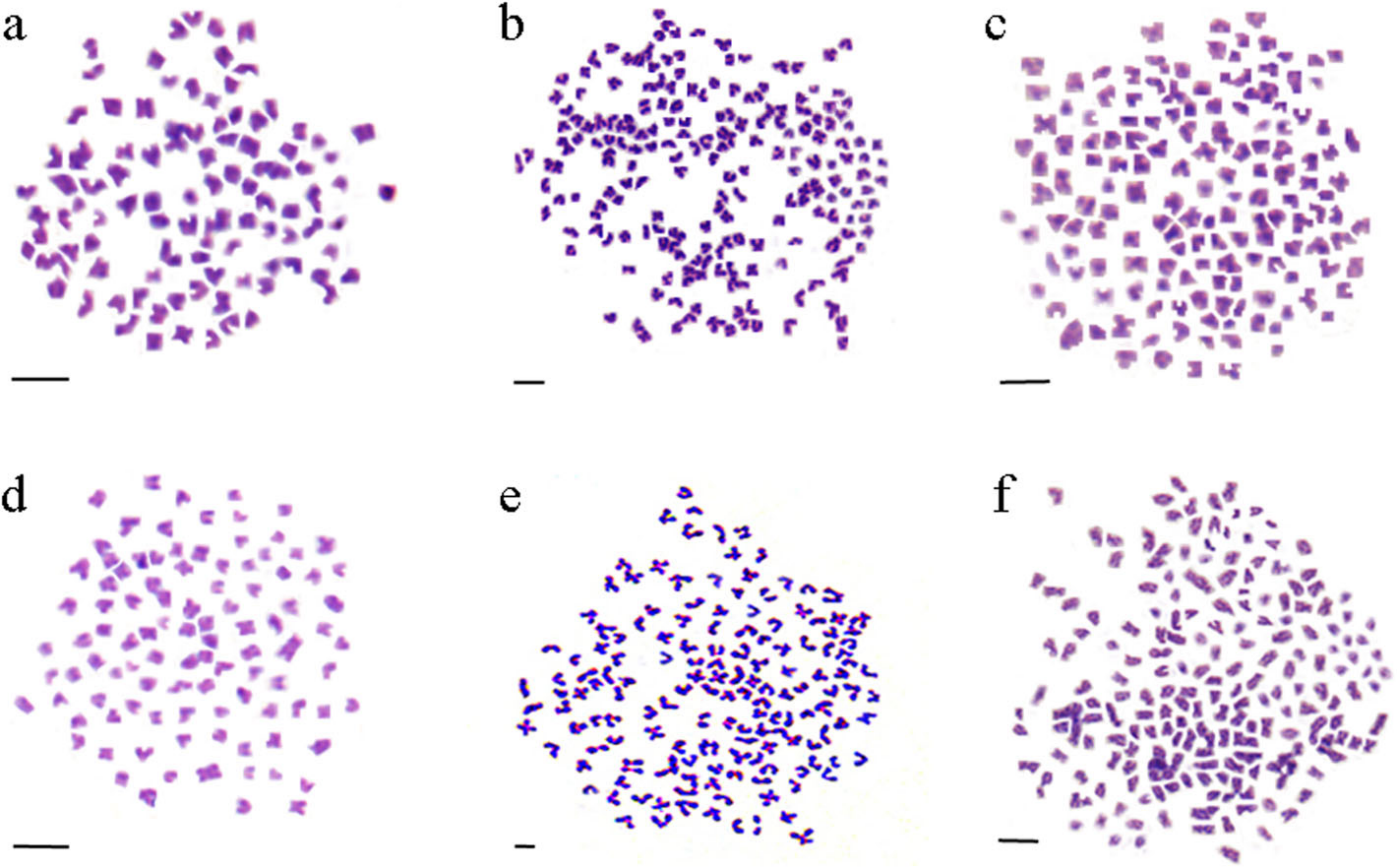
Chromosome spreads at metaphase in RCC, 4nRR, 3nRR, 2nF_1_, 3nF_1_ and  4nF_1_. **a**: The 100 chromosomes of RCC; **b**: The 200 chromosomes of 4nRR; **c**: The 150 chromosomes of 3nRR; **d**: The 100 chromosomes of 2nF_1_; **e**: The 150 chromosomes of 3nF_1_; **f**: The 200 chromosomes of 4nF_1_; bar = 5 μm

**Fig. 4:**
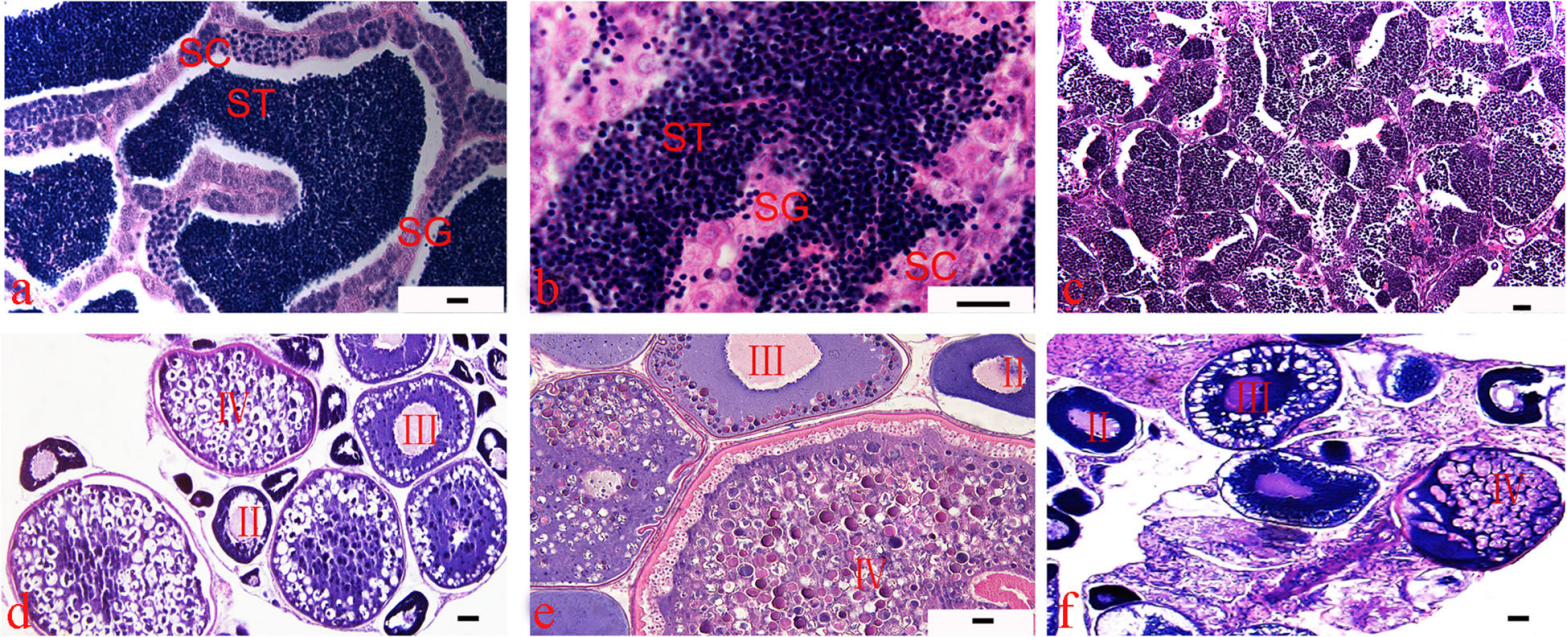
Micrographs of the testes and ovaries of RCC, 3nRR and 4nRR. **a**: Micrographs of testis from RCC; **b**: Micrographs of testis from 4nRR; **c**: Micrographs of testis from 3nRR; **d**: Micrographs of ovary from RCC; **e**: Micrographs of ovary from 4nRR; **f**: Micrographs of ovary from 3nRR; SG: spermatogonia; SC: spermatocyte; ST: spermatid; II: stage II oocyte; III: stage III oocyte; IV: stage IV oocyte; Bars = 50 μm

3nRR were generated by crossing female RCC and male 4nRR during the breeding season (Figs. 1a, b and c, 2 and 3a, b and c; Table 1). Testes of RCC and 4nRR (Fig. 4a, b) contained spermatogonia (SG), spermatocytes (SC) and a large number of mature spermatid (ST), whereas the mature sperm was not observed in 3nRR (Fig. 4c). Ovaries of RCC, 4nRR and 3nRR contained second, third and fourth phase oocytes (Fig. 4d, e, f). These results indicated that all ovaries, and RCC and 4nRR testes were fertile whereas 3nRR testes were sterile.

**Table 1:** Examination of chromosome number of RCC, 4nRR, 3nRR, 2nF_1_, 3nF_1_ and 4nF_1_

Fish type	No. of metaphase	Distribution of chromosome number					
		< 100	100	< 150	150	< 200	200
RCC	200	15	185				
4nRR	200					26	174
3nRR	200			17	183		
2nF_1_	200	18	182				
3nF_1_	200			22	178		
4nF_1_	200					34	166

**Fig. 5:**
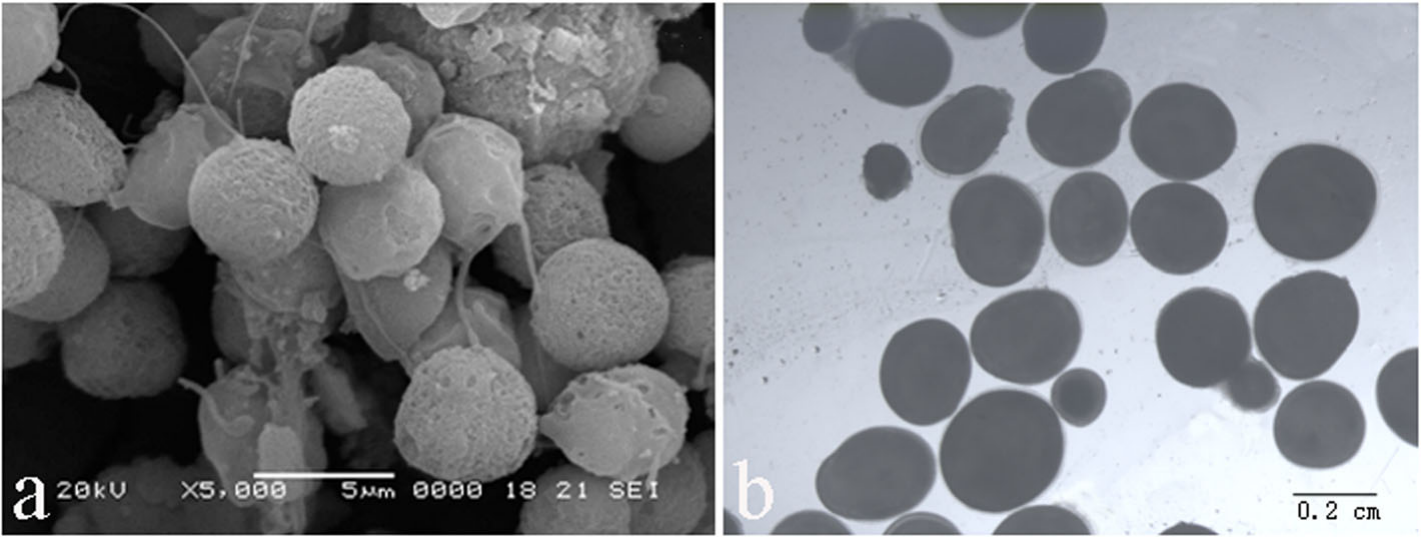
Spermatozoa and eggs of 3nRR. **a**: Abnormal spermatozoa produced by 3nRR males; **b**: Different sizes of eggs collected from 2-year-old 3nRR females

Eggs and water-like semen were collected during the reproductive season from two years old males and females of 3nRR, respectively (Fig. 5). Ploidy levels of the offspring resulting from a cross of female 3nRR and male RCC (Fig. 1c, d, e, f, g) were determined by measuring the chromosome number (Fig. 3d, e, f; Table 1). These analyses showed that female 3nRR produced different sizes of eggs.

### Transcriptome sequencing and sequence alignment

Optical density (OD) ratio A260/A280 and RNA integrity numbers (RINs) of the RNA in 12 samples (Table 2) were 2.1 and 8.0-8.8, respectively (Additional file 1). These results indicate that all samples were free from contamination and their quality met the requirements for transcriptome sequencing.

**Table 2:** Sample information

Sample No.	Sample type
FRCC-1	RCC-1 (female parent)
FRCC-2	RCC-2 (female parent)
FRCC-3	RCC-3 (female parent)
M4nRR-1	4nRR-1 (male parent)
M4nRR-2	4nRR-2 (male parent)
M4nRR-3	4nRR-3 (male parent)
F3nRR-1	F_1_ (male) (RCC×4nRR)-1
F3nRR-2	F_1_ (male) (RCC×4nRR)-2
F3nRR-3	F_1_ (male) (RCC×4nRR)-3
M3nRR-1	F_1_ (female) (RCC×4nRR)-1
M3nRR-2	F_1_ (female) (RCC×4nRR)-2
M3nRR-3	F_1_ (female) (RCC×4nRR)-3

RNA-seq from gonadal tissue samples of autotriploid fish and their parents was performed by Illumina. RNA-Seq results are presented in Tables 3 and 4. Number of clean reads from the 12 RNA-seq libraries ranged from 39,624,312 to 50,588,484. All clean reads were then aligned to the RCC genome sequences using HISAT2 software. Mapped genome reads ranged from 24,237,536 to 42,474,296, genome map rates ranged from 59.79 to 91.55 %, and unique match rates ranged from 57.84 to 85.93 %.

**Table 3:** Summary of the RNA-Seq data collected from FRCC, M4nRR, F3nRR and M3nRR

Sample name	Raw reads	Clean reads	Clean bases	Q20 (%)	Q30 (%)	GC content (%)
FRCC-1	42,334,070	42,252,766	6.31G	97.83	93.94	48.99
FRCC-2	39,685,218	39,624,312	5.92G	97.81	93.88	48.20
FRCC-3	46,465,082	46,395,874	6.94G	97.79	93.75	48.18
M4nRR-1	42,549,848	42,506,792	6.34G	97.63	93.34	45.99
M4nRR-2	42,757,282	42,709,522	6.36G	97.65	93.46	46.84
M4nRR-3	40,576,492	40,538,356	6.05G	97.84	93.80	45.67
F3nRR-1`	40,938,734	40,848,106	6.07G	97.17	92.48	46.81
F3nRR-2	50,703,558	50,588,484	7.54G	97.43	93.03	47.14
F3nRR-3	48,905,962	48,796,080	7.28G	97.48	93.14	47.23
M3nRR-1	45,040,604	44,995,054	6.70G	97.72	93.57	46.43
M3nRR-2	44,702,364	44,659,854	6.67G	98.09	94.25	45.66
M3nRR-3	45,015,326	44,976,210	6.72G	97.75	93.57	45.70

**Table 4:** Summary of clean reads mapped from FRCC, M4nRR, F3nRR and M3nRR to the reference genome

Sample name	Total reads	Total mapped	Multiple mapped	Uniquely mapped
FRCC-1	42,252,766	38,590,536 (91.33 %)	2,470,549 (5.85 %)	36,119,987 (85.48 %)
FRCC-2	39,624,312	36,222,309 (91.41 %)	2,283,305 (5.76 %)	33,939,004 (85.65 %)
FRCC-3	46,395,874	42,474,296 (91.55 %)	2,608,537 (5.62 %)	39,865,759 (85.93 %)
M4nRR-1	42,506,792	25,481,741 (59.95 %)	791,414 (1.86 %)	24,690,327 (58.09 %)
M4nRR-2	42,709,522	26,274,265 (61.52 %)	1,570,067 (3.68 %)	24,704,198 (57.84 %)
M4nRR-3	40,538,356	24,237,536 (59.79 %)	749,604 (1.85 %)	23,487,932 (57.94 %)
F3nRR-1	40,848,106	28,790,170 (70.48 %)	1,218,302 (2.98 %)	27,571,868 (67.50 %)
F3nRR-2	50,588,484	35,839,842 (70.85 %)	1,526,075 (3.02 %)	34,313,767 (67.83 %)
F3nRR-3	48,796,080	34,635,763 (70.98 %)	1,489,678 (3.05 %)	33,146,085 (67.93 %)
M3nRR-1	44,995,054	31,933,903 (70.97 %)	1,010,710 (2.25 %)	30,923,193 (68.72 %)
M3nRR-2	44,659,854	31,782,067 (71.16 %)	982,486 (2.20 %)	30,799,581 (68.96 %)
M3nRR-3	44,976,210	31,860,677 (70.84 %)	1,043,971 (2.32 %)	30,816,706 (68.52 %)

### Identification of Differentially Expressed Transcripts (DETs)

**Fig. 6:**
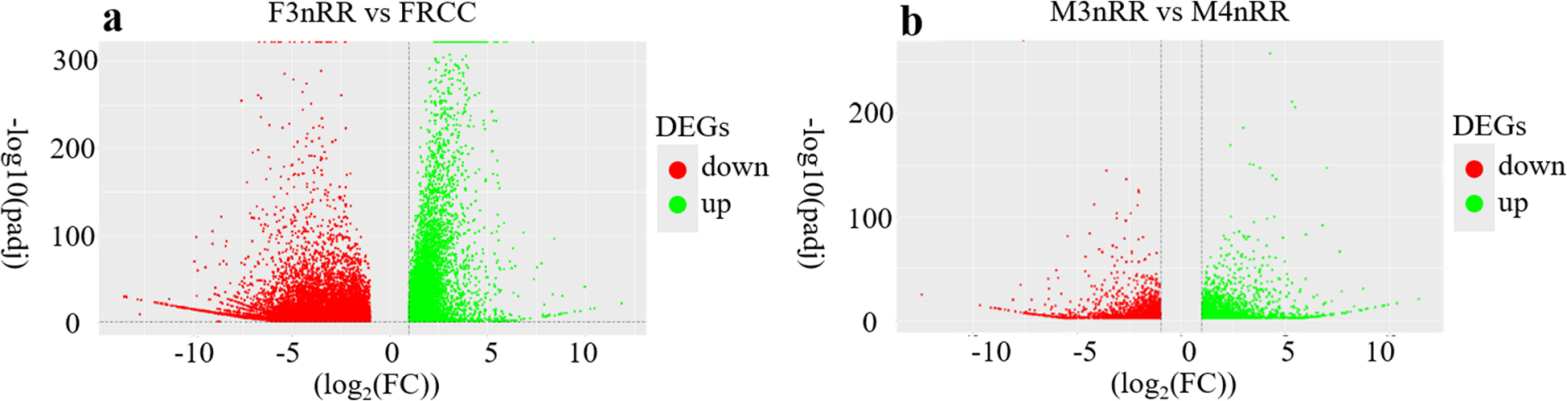
Volcano plot for transcript differential expression. **a**: F3nRR vs. FRCC; **b**: M3nRR vs. M4nRR. Transcripts with FDR < 0.05 and ratio of FPKMs of the two samples > 2 were considered to be differentially expressed transcripts. The red region shows significantly up-regulated transcripts, whereas the green region shows down-regulated transcripts

Analysis of F3nRR and FRCC showed that a total of 13,467 DETs were downregulated whereas 6,688 DETs were up-regulated (Fig. 6a). DETs between F3nRR and FRCC included forkhead box L2 (*FOXL2*), LIM homeobox 8 (*LHX8*), lysine acetyltransferase 8 (*KAT8*), BCL2 apoptosis regulator (*BCL2*), doublesex and mab-3 related transcription factor 1 (*DMRT1*), ovarian serine protease (*OSP*) and CCM2 scaffold protein (*CCM2*). Analysis of M3nRR and M4nRR showed that a total of 1,886 DETs were downregulated and 2,221 DETs were up-regulated (Fig. 6b). DETs between M3nRR and M4nRR included septin 12 (*SEPT12*), ATPase copper transporting beta (*ATP7B*), CF transmembrane conductance regulator (*CFTR*), cAMP responsive element modulator (*CREM*), cytochrome P450 family 26 subfamily B member 1 (*CYP26B1*), EF-hand calcium binding domain 2 (*EFCAB2*) and inhibitor of kappa light polypeptide gene enhancer in B-cells and kinase complex-associated protein (*IKBKAP*).

### GO and KEGG enrichment analysis of DETs

**Fig. 7:**
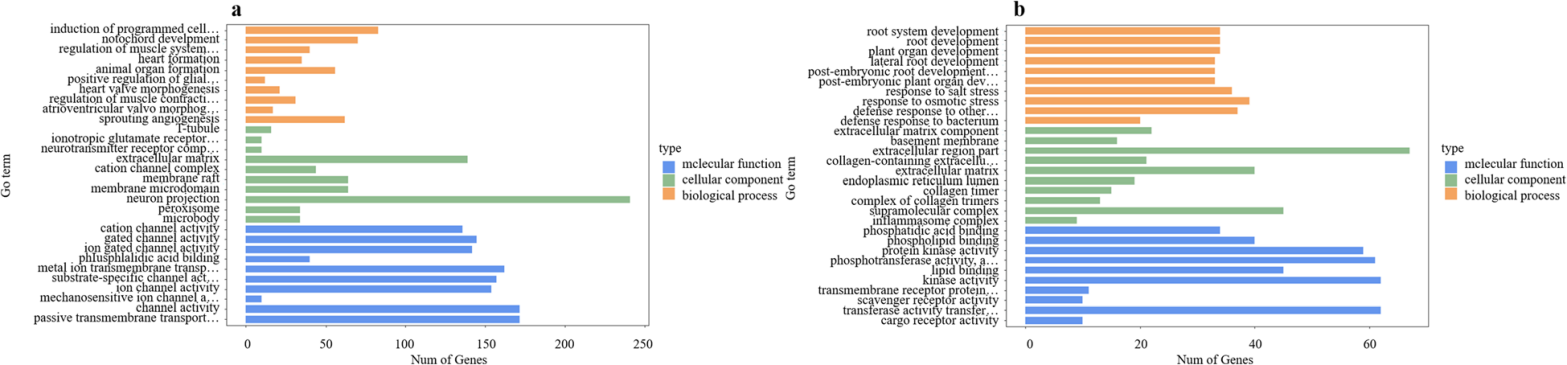
Gene Ontology (GO) functional classification of differentially expressed transcripts (DETs). **a**: F3nRR vs. FRCC; **b**: M3nRR vs. M4nRR. The three terms are presented on the x-axis shows and the proportion of DETs corresponding to each subcategory is presented on the y-axis

GO enrichment analysis of the biological process, cellular component and molecular function categories yielded 242, 38 and 51 terms, respectively, for F3nRR vs. FRCC, and 223, 28 and 29 for M3nRR vs. M4nRR group. (Additional files 2 and 3). The most-enriched GO-terms for F3nRR vs. FRCC group were “induction of programmed cell death” in the biological process category, “neuron projection” in the cellular component category, and “channel activity” and “passive transmembrane transporter activity” in the molecular function category. The most-enriched GO-terms for M3nRR vs. M4nRR group were “extracellular region part” in the cellular component category; “kinase activity” and “transferase activity, transferring phosphorus-containing groups” in the molecular function category; and “response to osmotic stress” in the biological process category (Fig. 7).

**Fig. 8:**
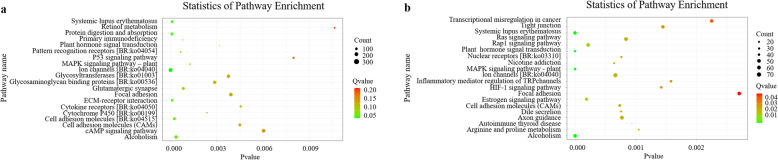
Statistics of Kyoto Encyclopedia of Genes and Genomes (KEGG) pathway enrichment analysis of the functional significance of DETs. **a**: F3nRR vs. FRCC; **b**: M3nRR vs. M4nRR. The abscissa represents P value, which decreases with increase in significance in enrichment level of differentially expressed transcripts in the pathway. The ordinate represents log10 (Q value), which increases with increase in significance of differentially expressed transcripts in the pathway

KEGG analysis of all DETs showed that 44 and 62 signaling pathways were enriched in the F3nRR vs. FRCC group and M3nRR vs. M4nRR group, respectively (Additional files 4 and 5). The top 20 most enriched KEGG pathways are shown in Fig. 8. The five most-enriched pathways in the F3nRR vs. FRCC group were “ion channels” (ko04040), “cAMP signaling pathway” (ko04024), “focal adhesion” (ko04510), “glycosaminoglycan binding proteins” (ko00536) and “glycosyltransferases” (ko01003). Moreover, several pathways implicated in female fertility of F3nRR were identified, including “MAPK signaling pathway - plant” (ko04016), and “p53 signaling pathway” (ko04115). The five most enriched pathways for the M3nRR vs. M4nRR group were “Ion channels” (ko04040), “rap1 signaling pathway” (ko04015), “ras signaling pathway” (ko04014), “alcoholism” (ko05034) and “axon guidance” (ko04360). Notably, four of the top 20 most-enriched pathways, “regulation of actin cytoskeleton” (ko04810), “calcium signaling pathway” (ko04020), “tight junction” (ko04530) and “cytokines and growth factors” (ko04052), play important roles in cellular processes such as differentiation, proliferation, migration and apoptosis, implying that they are potentially involved in male sterility of M3nRR.

### Hub genes related to the fertility in 3nRR were identified

**Fig. 9:**
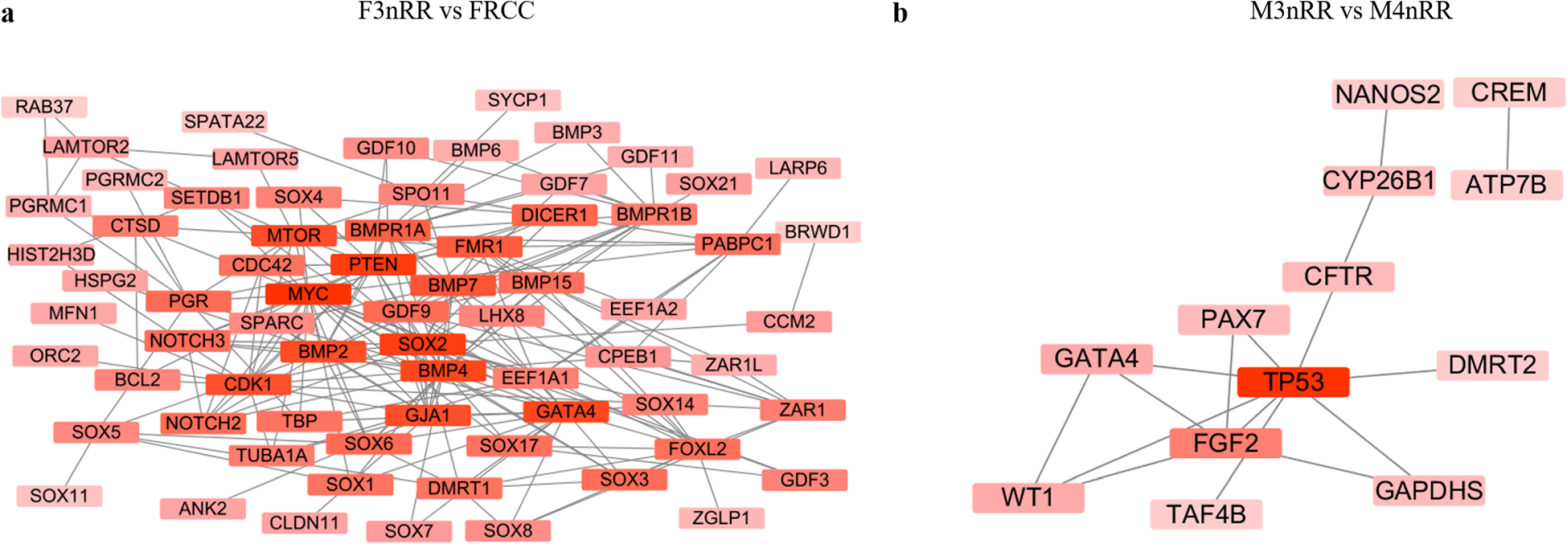
Protein-protein interaction network of fertility-related genes. **a**: F3nRR vs. FRCC; **b**: M3nRR vs. M4nRR. Genes with higher degree value are presented in deep red color

Eighty genes out of the DETs identified in the F3nRR vs. FRCC group related to female fertility were identified by literature supported searching (Additional file 6). On the other hand, 25 genes out of the DETs in the M3nRR vs. M4nRR group are implicated in male sterility (Additional file 7). To further identify hub genes associated with 3nRR fertility, PPI of the fertility-related genes was constructed using STRING tool and analysis was carried out using Cytoscape software. After analysis of PPI network of female fertility-related genes, 6 genes with the interaction degrees more than 15 were screened as hub genes (Fig. 9a, Additional file 8). Furthermore, PPI of male sterility-related genes showed that 2 hub genes, with degrees more than 5 showed strong interaction with other node proteins (Fig. 9b, Additional file 9).

### RT-qPCR verification

**Fig. 10:**
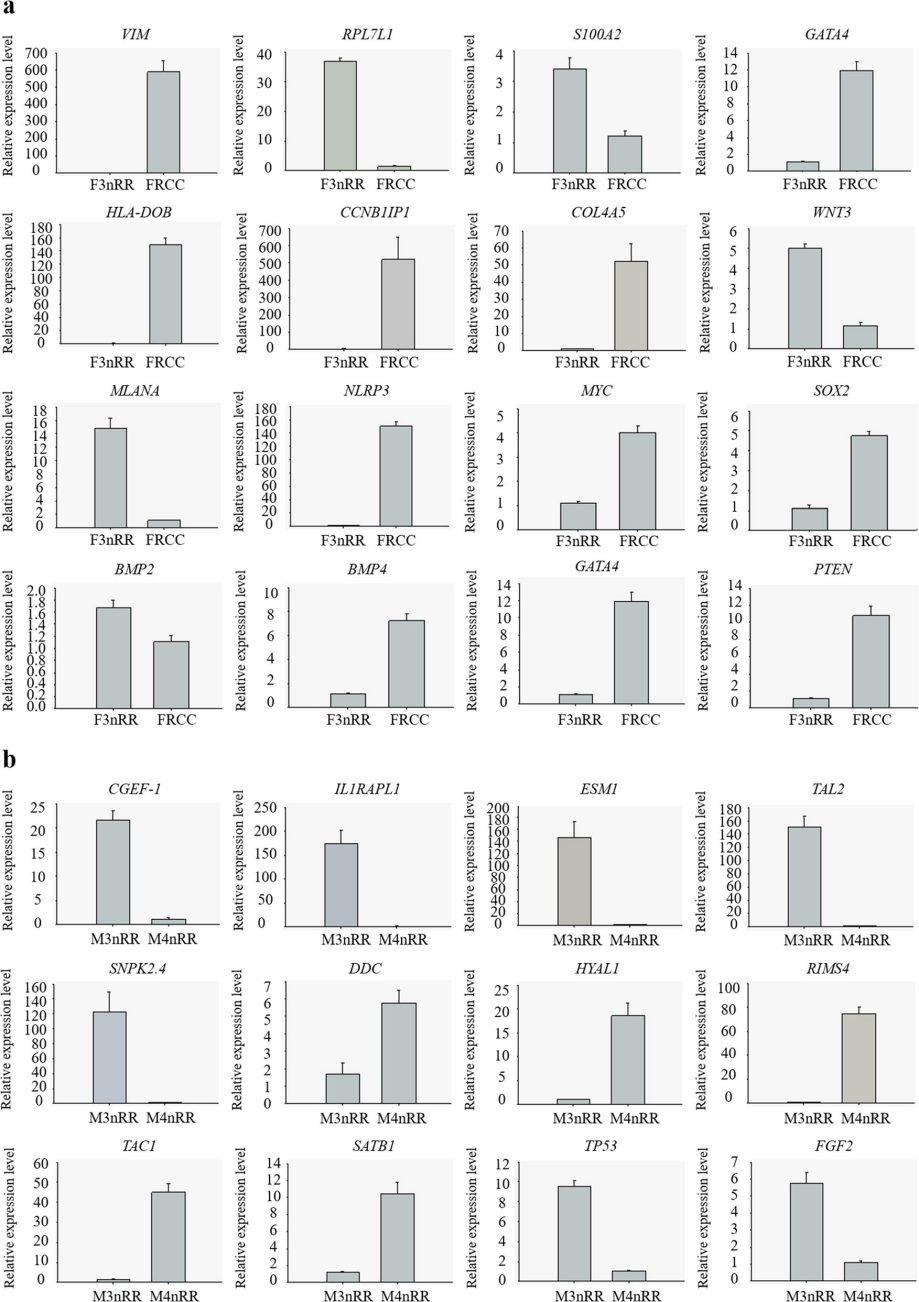
qPCR analysis of selected DETs. **a**: F3nRR vs. FRCC; **b**: M3nRR vs. M4nRR. Data represent the means ± SD, *n* = 3 independent experiments. *p < 0.01 versus control

To verify RNA-Seq results, twenty-eight DETs were chosen for validation by RT-qPCR. Among the 28 DETs, 6 DETs and 7 DETs were up-regulated in the F3nRR vs. FRCC and M3nRR vs. M4nRR groups, respectively; whereas 10 DETs and 5 DETs were down-regulated in the F3nRR vs. FRCC and M3nRR vs. M4nRR groups, respectively (Fig. 10). Expression profiles of the twenty DEGs obtained by RT-qPCR and RNA-Seq were similar, implying that RNA-Seq results were reliable.

## Discussion

Triploid animals are usually sterile and cannot form triploid populations. However, previous studies have been reporting contradicting results. Xiao et al. [25] reported that triploid *Carassius auratus* in Dongting water system produces normal gametes. Hu et al. [26] reported that female autotriploid hybrids (3nAUT) generated by crossing females of *Carassius auratus* red var. with males of autotetraploid fish produced mature eggs. However, male 3nAUT showed abnormal gonadal development and could not produce mature sperm. In the present study, 3nRR was generated by crossing female RCC and male 4nRR. Three different ploidy offspring were then obtained by hybridization of female 3nRR and male RCC. The female 3nRR offspring were fertile, whereas male 3nRR were sterile. However, only a few studies have explored the molecular mechanisms modulating fertility of the autotriploid of *Carassius auratus*. In this study transcriptome analysis was used to explore the molecular mechanisms associated with poor fertility in 3nRR. Eight fertility-related hub genes of 3nRR were identified through GO and KEGG enrichment analyses, and previous published literature.

### Candidate hub genes related to male sterility of 3nRR were identified

Hub genes identified in the M3nRR vs. M4nRR group included several genes involved in male sterility, such as the tumor protein p53 (*TP53*) and fibroblast growth factor 2 (*FGF2*).

*TP53*, also known as *P53* is a transcriptional regulator and tumor suppressor implicated in spermatogenesis [27]. In vertebrates, partial or complete impairment of *P53* expression causes disordered meiotic divisions, which in turn causes spermatogenesis defects [28, 29]. *P53* mRNA and protein levels are downregulated in the testis of *P53* promoter-chloramphenicol acetyltransferase (CAT)-harboring mice, indicating its important role in development of spermatocytes [30]. In addition, *TP53* codon 72 polymorphism in mice is involved in meiosis, implying that it plays a critical role in spermatogenesis [31]. In human, *P53* gene polymorphism is higher in infertile men compared with fertile men, implying that it may affect germ cell apoptosis and increase risk of male infertility [29, 32]. In the current study, analysis of expression levels of the *TP53* gene showed significantly different expression levels between M3nRR and M4nRR. This finding implies that *TP53* may disrupt meiosis during spermatogenesis in the male 3nRR fish causing sterility.

Fibroblast growth factor 2 (*FGF2*) plays essential functions in regulation of spermatogenesis and sperm physiology [33]. A study using a human model reported presence of *FGF2* and *FGFR*s in testis and sperm, which are related with human spermatogenesis and sperm motility [34]. Furthermore, incubation of human sperm with recombinant *FGF2* (r*FGF2*) causes an increase in number of motile cells, implying that the gene is involved in sperm motility [35]. In mouse, knock out of *FGF2* induces impaired sperm production and is associated with alterations in sperm morphology and function [36]. In this study, *FGF2* was significantly upregulated in M3nRR vs. 4nRR. High expression levels of the gene can cause abnormal shaping of the normal sperm, which resulted in male 3nRR sterility.

### Candidate hub genes related to female fertility of 3nRR were identified

Six hub genes associated with female fertility were identified in the F3nRR vs. F4nRR group including MYC proto-oncogene, bHLH transcription factor (*MYC*), SRY-box transcription factor 2 (*SOX2*), bone morphogenetic protein 4 (*BMP4*), GATA binding protein 4 (*GATA4*), phosphatase and tensin homolog (*PTEN*) and bone morphogenetic protein 2 (*BMP2*).

*MYC* gene encodes the *MYC* transcription factor which is involved in cell proliferation and gametogenesis [37]. In *Xenopus*, *C-MYC* was detected in oocytes, indicating that it plays a role in oogenesis [38]. In *Drosophila, MYC* was involved in distribution of CTPsyn in follicle cells, implying that it plays a role in synthesizing nutrients for the developing oocytes [39]. In addition, a previous study reported that *MYC* plays an important role in regulation of mitochondrial biogenesis in *Drosophila* ovary, indicating that the gene is involved in oocyte development [40]. In *Larimichthys crocea*, *Lc-cMYC* had different expression patterns in oocytes at various stages of development, implying that it plays an essential role in oogenesis [41]. In the current study, 3nRR ovary showed low expression level of *MYC* which may inhibited formation of oogenesis defects during triploidization, resulting in production mature eggs.

*SOX* is an ancient gene family involved in oogenesis [42]. *Sox* genes have been explored in many organisms and can be classified into ten subgroups (A-J) [43]. In *Agasicles hygrophila*, *AhDichaete* and *AhSox3* expression levels are significantly high in ovary, indicating that it plays a vital regulatory role in during ovarian development and oogenesis [44]. In *Misgurnus anguillicaudatus*, *MaSOX3* is abundant in primary oocytes and previtellogenic oocyte cells, indicating that *MaSox3* gene is involved in ovarian development [45]. In *Paramisgurnus dabryanus*, *SOX4* was detected in the ovary, showing that it plays an important role during ovarian development [46]. In mouse, expression of *SOX2* is required for establishment and maintenance of the oocyte cell [47]. In our study, *SOX2* was identified in F3nRR vs. FRCC, with lower expression level in F3nRR gonad compared with the expression level in FRCC gonad. This finding shows that *SOX2* may be an important factor in normal ovarian development of F3nRR.

Bone morphogenetic proteins (BMPs) are belonging to the transforming growth factor-β superfamily of proteins, and they appear to be highly conserved [48]. A previous study reports that BMPs play a role in regulation of ovarian follicular development [49]. *BMP1*, *BMP6* and *BMP15* are implicated in ovarian development [50–52]. In *Xenopus laevis*, *BMP2* gene is highly expressed during oogenesis, implying that it is an important factor in ovarian development [53]. In mouse, *BMP4* regulates the number of oocytes, suggesting its role in the process of oogenesis [54]. Roles of *BMP2* and *BMP4* as important factors in survival and development of bovine secondary follicles were recently reported [55]. In this study, *BMP2* and *BMP4* genes were differentially expressed between F3nRR and FRCC, indicating that they may be involved in ovarian development.

GATA4, a member of the GATA-binding family, is highly expressed in ovarian granulosa cells [56, 57]. A previous study reported that the gene is involved in regulation of ovarian development [58]. *GATA4* and *GATA6* knockout female mice exhibited infertility due to disrupted formation of ovaries [59]. *GATA4* deletion resulted in a sterile female mice phenotype attributed to drastic reduction in number of developing follicles [60]. The mRNA for *GATA4* has been reported in human ovary implying that *GATA4* plays a role in ovarian folliculogenesis [57]. *GATA4* was identified through transcriptome analysis in this study. *GATA4* was differentially expressed in F3nRR vs. FRCC, indicating that plays important roles in ovarian development of 3nRR.

Phosphatase and tensin homolog (PTEN) protein has phosphatase activity and belongs to protein-tyrosine phosphatase superfamily [61]. PTEN is a negative regulator of PI3K-Akt signaling pathway which is involved in growth of eggs [62]. Deletion of *PTEN* from oocytes affects mouse fertility by interrupting oocyte growth [63]. PTEN signaling pathway associated with ovarian follicle development has been reported in human [64]. In *Crassostrea gigas*, *PTEN* is involved in insulin pathway in gonads and plays a critical role in reproduction [65]. In *Drosophila*, loss of *PTEN* is related to IIS/mTORC1 signalling, which is important for oogenesis [66]. In the present study, F3nRR showed significantly low expression levels of *PTEN*, which may have caused F3nRR normal fertility through regulation of oogenesis.

## Conclusions

The autotriploid *Carassius auratus* (3nRR, 3n = 150) is generated from *Carassius auratus* red var. (RCC, 2n = 100) (♀) and autotetraploid *Carassius auratus* (4nRR, 4n = 200) (♂), of which the female 3nRR can produce mature gametes, whereas the male 3nRR cannot. In addition, we produced diploid (2nF_1_, 2n = 100), triploid (3nF_1_, 3n = 150) and tetraploid (4nF_1_, 4n = 200) hybrids in the F_1_ generation by crossing females of 3nRR with males of RCC, which further indicated that female 3nRR were fertile. Gonadal transcriptome reveals 6 hub genes (*MYC*, *SOX2*, *BMP4*, *GATA4*, *PTEN* and *BMP2*) were involved in the female fertility of the female 3nRR, and 2 hub genes (*TP53* and *FGF2*) were involved in the male sterility of the male 3nRR. The obtained data reveals novel candidate genes for the fertility in the autotriploid fish and also extends an understanding of the molecular aspects of fertility in triploid fish.

## Methods

### Animals and crosses

One year old RCC and one year old 4nRR (F_11_) were fed in the State Key Laboratory of Developmental Biology of Freshwater Fish, Hunan Normal University, China. Hybrids (3nRR) of RCC (♀) × 4nRR (♂) were generated in May 2018. All fish were maintained in open pools (0.067 ha) with suitable pH (7.0-8.5), water temperature (22–24℃), dissolved oxygen content (5.0–8.0 mg/L) and adequate forage. All dissections were performed under MS-222 anaesthesia (100 mg/L; Sigma-Aldrich).

### Gonadal histologic analysis

Ploidy levels of the fish (RCC, 4nRR and 3nRR) were estimated using a flow cytometer (Gallios Flow Cytometer, Beckman Coulter). Blood was collected from the caudal vein using heparinized syringes. Samples were then resuspended in 4,6-Diamidino-2-Phenylindole solution (Sigma-Aldrich) for 10 min. DNA content was compared with that of RCC per sample. Gonadal tissues of two years old female RCC, male 4nRR and 3nRR were fixed in Bonn’s liquid and then dehydrated using graded series of alcohol, cleared with xylene, embedded in paraffin wax and cut into 5–8 μm sections. The sections were placed on slides, stained with hematoxylin and eosin, and viewed under a light microscope.

### Gamete phenotypes and egg ploidy detection

The water-like semen and mature eggs of two years old 3nRR were sampled for morphological examination. The female 3nRR produced different sized eggs. To determine the egg ploidy, mature eggs were used in vitro fertilization of the RCC haploid sperm and then viable offspring (2nF_1_, 3nF_1_ and 4nF_1_) were generated. Ploidy of these offspring was detected by chromosome counts.

### Preparation of chromosome spreads

For ploidy level analysis, chromosome counts were carried out using kidney tissues from 10 individuals each of RCC, 4nRR, 3nRR, 2nF_1_, 3nF_1_ and 4nF_1_ at eight months of age following a previously described method [67]. 200 metaphase chromosome spreads (20 spreads per sample) were analyzed for each type of fish. Each preparation was examined under 3330× magnification with an oil immersion lens.

### Sample collection and preparation for transcriptomic sequencing

A total of 3 females RCC (FRCC), 3 males 4nRR (M4nRR), 3 males 3nRR (M3nRR) and 3 females 3nRR (F3nRR) were acquired at 24 months. Fish were anesthetized before surgical removal of tissues. Gonadal tissues were harvested from FRCC, M4nRR, F3nRR and M3nRR after euthanasia.

### RNA extraction and sequencing

Total RNA was extracted from gonads of RCC, 4nRR and 3nRR using TRIzol reagent (Takara, Beijing, China) according to the instructions. RNA integrity (RNA integrity score ≥ 7.0) was checked on the bioanalyzer 2100 system (Agilent, Palo Alto, CA) and RNA quantity was measured using NanoDrop 2000 (Thermo, Waltham, MA, USA). A total of 12 libraries from FRCC, M4nRR, F3nRR and M3nRR groups were sequenced. In summary, mRNA was purified and broken into short fragments. Then, reverse transcription, cDNA synthesis and cluster generation were performed. RNA-Seq libraries were then sequenced on Illumina Hiseq2500 platform. The sequenced data are publicly available at the NCBI (PRJNA694292).

### Differential expressed transcripts (DETs) and profiling of potential fertility-related genes

After sequencing, clean reads were acquired by removing adapters and low-quality reads using fastp software (Version 0.20.0). The clean reads quality was assessed with FastQC software (Version 0.11.9). The clean reads of the libraries were aligned to the published RCC reference genome (https://bigd.big.ac.cn/search?dbId=gwh&q=GWHAAIA00000000) using HISAT2 tool (Version 2.1.0). To calculate gene expression level, we used fragments per kilobase per million mapped fragments (FPKM) method. Differentially expressed transcripts (DETs) analysis of F3nRR vs. FRCC, and M3nRR vs. M4nRR was performed using DEGSeq2 R package (Version 1.28.1). Transcripts having a fold change (FC) > 2 and a false discovery rate (FDR) < 0.05 were considered as DETs. To further explore these DETs, Gene ontology (GO) and Kyoto Encyclopedia of Genes and Genomes (KEGG) enrichment analysis were performed using clusterProfiler (Version 3.6.0) with *p* < 0.05. Moreover, fertility-related DETs were screened following GO, KEGG enrichment analyses and published literature. STRING database (https://string-db.org/) to construct protein-protein interaction (PPI) networks to explore protein relationships among the fertility-related DETs. Hub genes were obtained based on the ranking order of connectivity degree by Cytoscape software [68].

### Quantitative real-time PCR verification

Ten significantly DETs (five up-regulated DETs and five down-regulated DETs) and eight important genes in this study were chosen for quantitative real-time (qRT) PCR to test the reliability of the F3nRR vs. FRCC and M3nRR vs. M4nRR transcriptome sequencing results. PrimeScript™ RT reagent kit (Takara, Dalian, China) was used to perform cDNA synthesis following the manufacturer’s instructions. Primer sequences for *β-actin* (the internal control gene) and these DETs are listed in Additional file 10. The 10-µl-volume qRT-PCR reaction mixture consisted of 5 µl SYBR Green qPCR Master Mix, 0.5 µl of 20 µM of each primer, 1 µl of cDNA (1:10 dilution) and 3 µL of nuclease-free water. qRT-PCR thermal cycle used was as follows: 95 °C for 2 min, 40 cycles of 95 °C for 15 s and annealing at 60 °C for 30 s. Three technical replicates were used for each biological sample in the qRT-PCR. Relative mRNA expression level was calculated by using the 2^−ΔΔCt^ method. Data were analyzed statistically using SPSS (v22.0) software (SPSS Inc., Chicago, IL, USA). Statistical significance was determined using Student’s t-test analysis.

## Supplementary Information

**Additional file 1: Table S1.** Quality test results of RNA.

**Additional file 2: Table S2.** Transcript classification based on gene ontology (GO) for DETs in F3nRR vs FRCC.

**Additional file 3: Table S3.** Transcript classification based on gene ontology (GO) for DETs in M3nRR vs M4nRR.

**Additional file 4: Table S4.** Transcript classification based on Kyoto Encyclopedia of Genes and Genomes (KEGG) for DETs in F3nRR vs FRCC.

**Additional file 5: Table S5.** Transcript classification based on Kyoto Encyclopedia of Genes and Genomes (KEGG) for DETs in M3nRR vs M4nRR.

**Additional file 6: Table S6.** Female fertility-related genes.

**Additional file 7: Table S7.** Male fertility-related genes.

**Additional file 8: Table S8.** Table of F3nRR vs FRCC of fertility-related gene in protein-protein interaction network.

**Additional file 9: Table S9.** Table of M3nRR vs M4nRR of fertility-related gene in protein-protein interaction network.

**Additional file 10: Table S10.** Sequences of primers used in this study.

## Data Availability

Raw sequence reads are available from the NCBI (PRJNA694292) (https://www.ncbi.nlm.nih.gov/bioproject/PRJNA694292) and the expression profiles of RNA-Seq data are included in Figshare (10.6084/m9.figshare.14561670.v1).

## Abbreviations

RCC: *Carassius auratus* red var
3nRR: Autotriploid *Carassius auratus*
4nRR: Autotetraploid *Carassius auratus*
FRCC: Female *Carassius auratus* red var
M4nRR: Male autotetraploid *Carassius auratus*
F3nRR: Female autotriploid *Carassius auratus*
M3nRR: Male autotriploid *Carassius auratus*
PPI: Protein-protein interaction
2nF_1_: Diploid hybrids of female autotriploid *Carassius auratus* × male *Carassius auratus* red var.;
3nF_1_: Triploid hybrids of female autotriploid *Carassius auratus* × male *Carassius auratus* red var
4nF_1_: Tetraploid hybrids of female autotriploid *Carassius auratus* × male *Carassius auratus* red var
SG: Spermatogonia
SC: Spermatocytes
ST: Spermatid
DETs: Differentially expressed transcripts
GO: Gene Ontology
KEGG: Kyoto Encyclopedia of Genes and Genomes
*TP53*: Tumor protein p53
*FGF2*: Fibroblast growth factor 2
*MYC*: MYC proto-oncogene, bHLH transcription factor
*SOX2*: SRY-box transcription factor 2
*BMP4*: Bone morphogenetic protein 4
*GATA4*: GATA binding protein 4
*PTEN*: Phosphatase and tensin homolog
*BMP2*: Bone morphogenetic protein 2
