# Supplementary material for: Formation of autotriploid Carassius auratus and its fertility-related genes analysis
Source: BMC Genomics. 2021 Jun 10;22:435. doi: 10.1186/s12864-021-07753-5 (PMC8191051; doi:10.1186/s12864-021-07753-5)
Supplement: Supplementary file 1 — Additional file 1: Table S1. Quality test results of RNA. [file 12864_2021_7753_MOESM1_ESM.docx]

| Sample name | RIN | 28S/18S | OD260/280 | QC  Evaluation | Amount  (µg) |
| --- | --- | --- | --- | --- | --- |
| FRCC-1 | 8.3 | 2.0 | 2.1 | A | 55.2 |
| FRCC-2 | 8.1 | 2.1 | 2.1 | A | 46.3 |
| FRCC-3 | 8.1 | 2.0 | 2.1 | A | 39.4 |
| M4nRR-1 | 8.2 | 2.1 | 2.1 | A | 43.2 |
| M4nRR-2 | 8.6 | 2.2 | 2.1 | A | 28.3 |
| M4nRR-3 | 8.0 | 2.0 | 2.1 | A | 41.8 |
| F3nRR-1` | 8.3 | 2.4 | 2.1 | A | 72.1 |
| F3nRR-2 | 8.2 | 2.0 | 2.1 | A | 44.9 |
| F3nRR-3 | 8.3 | 2.0 | 2.1 | A | 36.4 |
| M3nRR-1 | 8.8 | 2.3 | 2.1 | A | 20.3 |
| M3nRR-2 | 8.5 | 2.0 | 2.1 | A | 26.8 |
| M3nRR-3 | 8.6 | 2.1 | 2.1 | A | 27.2 |

Additional file 1: Table S1. Quality test results of RNA.
