# Supplementary material for: Formation of autotriploid Carassius auratus and its fertility-related genes analysis
Source: BMC Genomics. 2021 Jun 10;22:435. doi: 10.1186/s12864-021-07753-5 (PMC8191051; doi:10.1186/s12864-021-07753-5)
Supplement: Supplementary file 10 — Additional file 10: Table S10. Sequences of primers used in this study. [file 12864_2021_7753_MOESM10_ESM.docx]

Additional file 10: Table S10. Sequences of primers used in this study.

| Gene name | Primer Sequence (5′ to 3′) |
| --- | --- |
| *RPL7L1*(Caur.EVM.07745-RA)  *S100A2*(Caur.EVM.28620-RA)  *GATA2*(Caur.EVM.36361-RA)  *WNT3*(Caur.EVM.31139-RA)  *MLANA*(Caur.EVM.37808-RA)  *COL4A5*(Caur.EVM.34835-RA)  *HLA-DOB(*Caur.EVM.24201-RA)  *VIM(*Caur.EVM.30470-RA)  *CCNB1IP1*(Caur.EVM.13868-RA)  *NLRP3*(Caur.EVM.09543-RA)  *MYC*(Caur.EVM.17341-RA)  *SOX2*(Caur.EVM.03022-RA)  *BMP2*(Caur.EVM.38198-RA)  *BMP4*(Caur.EVM.06347-RA)  *GATA4*(Caur.EVM.38286-RA)  *PTEN(*Caur.EVM.11833-RA)  *TAL2*(Caur.EVM.22800-RA)  *SNRK2.4*(Caur.EVM.17139-RA)  *CGEF-1*(Caur.EVM.36741-RA)  *IL1RAPL1*(Caur.EVM.11842-RA)  *ESM1*(Caur.EVM.11853-RA) | F: ATGCCACCACCAAAGAAC  R: GCAACATAGGGCTCCACA  F: CGACTCTAACCCAGCCAAAC  R: AGGTCTCCTCGCAAAGCA  F: GGTAAGGGACGCTGGGAAGT  R: CCGCCACAGAGGAGTTGATG  F: AATGTGCGGCTGTGACTC  R: CAGGTCTCGTTCTGTGGG  F: CCTGGGCTGCTGGTATTA  R: CCTGTGCTGACCCGCTTT  F: CATTGCCACCTCCTTGTT  R: GAATGCGACTTTGCTTGG  F: CCTTCAATAAAGTCGTGGAT  R: AGCGGACTGAGCATTAGG  F: TGAACTCGCCTTCCTAAA  R: CATTGGACTCCTGCTTGG  F: AAAGTTGAGTGGGTTTGCG  R: TTGTATTGCTCTGAGGGTG  F: AAAGGACCTACTTATTGC  R: AAGATTACACTCTGCCACT  F: GGATGCCTGGTTGCTTAT  R: GTCCGTGCTGCTGGTTAT  F: CAACTCTTCGGGAAACAACC  R: TGGGCATAACTGTCCATCCT  F: CCAAGGAGCACTATGGGAAAG  R: CGGCAGGAACGGATGTAAG  F: GAATGCCGATGGTCCTTT  R: TCCTCCTCTGGGATGCTG  F: GACGGGTTGGTTTGTCCTG  R: CACTGATGCTCGCTGGTTCT  F: GATGATGTAGTGCGATTC  R: AGTTACTCCCTTCTTGTCT  F: GCGGAAACTCATCCCCACA  R: TCCAGCAGAGTCACCAAGA  F: GGATTCGGCTCCGTCTTT  R: CCAGGGTCTCCGTGTTGA  F: GACATCAGTCAGGGTTTG  R: TACATCATCCTTGCCACA  F: AGGTTTGGGAGCCATTCT  R: TCCACTTCCTGGTAGTTCAT  F: CAGGTCTCGTTCTGTGGG  R: GCATCCGCAGTCATCCTG |
| Gene name | Primer Sequence (5′ to 3′) |
| *HYAL1*(Caur.EVM.06470-RA)  *RIMS4*(Caur.EVM.06490-RA)  *DDC*(Caur.EVM.18965-RA)  *TAC1*(Caur.EVM.20101-RA)  *SATB1*(Caur.EVM.15869-RA)  *TP53*(Caur.EVM.34661-RA)  *FGF2*(Caur.EVM.02939-RA)  *β-actin* | F: TCCTGCTCTGGAGATGAA  R: ATCCTGGCGTAGGTAAAG  F: TCGGAGGGAAATCTGAAC  R: TGTCTGCCCACAAACTGA  F: CCTCATAAATGGCTCCTG  R: AGAACAAACTTGCCTACC  F: CCGACCTCATCAGTTCAT  R: CCGACCTCATCAGTTCAT  F: GCTGCGGTGCCAGTGATT  R: AGCCGTTGGTCCCGTCTT  F: TTACTCCTATTGCTGTCA  R: TATCTTTCCTTACCCTTC  F: CAGGGTTCTGATCCGTGGGT  R: TCCGCCGTTCGGTGTTTT  F: GCCCTGCCCCATGCCATCCT  R: AGTGCCCATCTCCTGCTCGA |
